# Recurrent Syncope upon Deglutition

**DOI:** 10.5334/jbsr.1584

**Published:** 2018-08-30

**Authors:** Sammy Tawk, Gauthier Desuter, Sanaa Jamali

**Affiliations:** 1 Cliniques univeristaires Saint-Luc, BE

**Keywords:** Deglutition syncope, Hyoid bone, Neck, Carotid

## Case Study

A 67-year-old man presented to the department of otorhinolaryngology (ENT) with the complaint of recurrent syncope upon deglutition since several months. He reported symptoms ranging from light headedness to syncope triggered by deglutition. He suffered from end stage renal disease with no other relevant past medical history.

Physical exam, carotid duplex ultrasound and electrocardiogram were unremarkable. No external compression of the larynx was noted during laryngoscopy. Before assuming that the patient actually suffered from deglutition syncope (DS) syndrome, a non-enhanced computed tomography (CT) of the neck was performed. It revealed right posterior tilting of the hyoid bone with its right greater horn abutting the distal right common carotid artery, just before its bifurcation (arrow in Figures A and B). Tilting of the hyoid bone and the thyroid cartilage was better demonstrated on three-dimensional image reconstruction (Figure C).

**Figure A:**
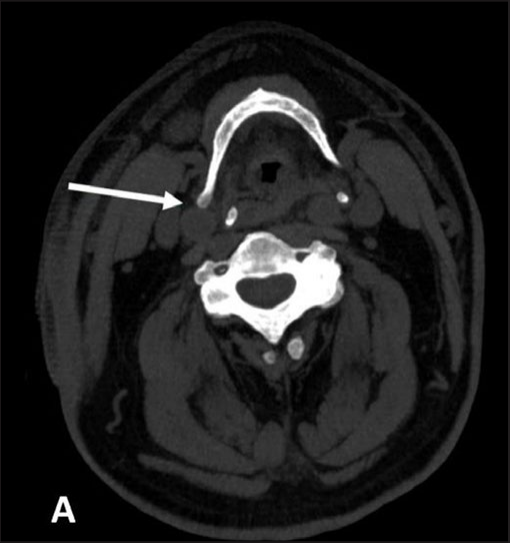
Reformatted non-enhanced oblique axial CT-scan image of the neck (soft tissue window) showing right posterior tilting of hyoid bone with its right greater horn (arrow) abutting the anterior wall of the distal right common carotid artery.

**Figure B:**
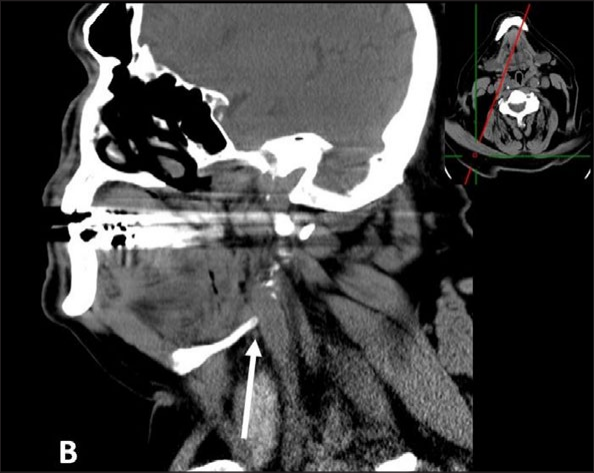
Reformatted non-enhanced oblique sagittal CT-scan image of the neck (soft tissue window) showing the greater horn of the hyoid bone (arrow) abutting the anterior wall of the distal right common carotid artery.

**Figure C:**
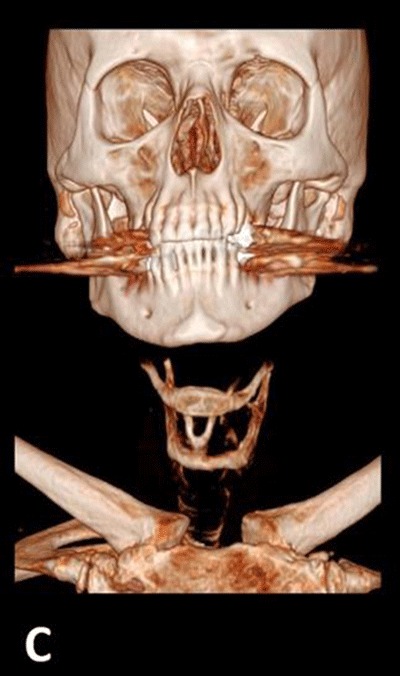
Three-dimensional (3D) volume rendering image of the neck CT-scan showing tilting of the hyoid bone and thyroid cartilage.

We assumed that ascension of the hyoid bone upon deglutition triggered carotid sinus stimulation responsible for the syncope.

Although uncommon, irritation/compression of the common/internal carotid artery by hyoid bone anomalies should be considered in the differential diagnosis of syncope, transient cerebral ischemia (TIA) and stroke, when no other causes are found [1]. The hyoid bone can directly compress the carotid artery or can incite local atherosclerotic plaque formation. When the syncope is triggered by deglutition, this phenomenon should be considered in the differential diagnosis, along with DS syndrome and postprandial hypotension.
